# Gaussian Mixture Models for Control of Quasi-Passive Spinal Exoskeletons

**DOI:** 10.3390/s20092705

**Published:** 2020-05-09

**Authors:** Marko Jamšek, Tadej Petrič, Jan Babič

**Affiliations:** 1 Laboratory for Neuromechanics and Biorobotics, Department of Automation, Biocybernetics and Robotics, Jožef Stefan Institute, 1000 Ljubljana, Slovenia; tadej.petric@ijs.si (T.P.); jan.babic@ijs.si (J.B.); 2 Jožef Stefan International Postgraduate School, Jamova cesta 39, 1000 Ljubljana, Slovenia

**Keywords:** pattern recognition, movement prediction, exoskeleton control, clutched elastic actuators

## Abstract

Research and development of active and passive exoskeletons for preventing work related injuries has steadily increased in the last decade. Recently, new types of quasi-passive designs have been emerging. These exoskeletons use passive viscoelastic elements, such as springs and dampers, to provide support to the user, while using small actuators only to change the level of support or to disengage the passive elements. Control of such devices is still largely unexplored, especially the algorithms that predict the movement of the user, to take maximum advantage of the passive viscoelastic elements. To address this issue, we developed a new control scheme consisting of Gaussian mixture models (GMM) in combination with a state machine controller to identify and classify the movement of the user as early as possible and thus provide a timely control output for the quasi-passive spinal exoskeleton. In a leave-one-out cross-validation procedure, the overall accuracy for providing support to the user was 86.72±0.86% (mean ± s.d.) with a sensitivity and specificity of 97.46±2.09% and 83.15±0.85% respectively. The results of this study indicate that our approach is a promising tool for the control of quasi-passive spinal exoskeletons.

## 1. Introduction

Exoskeletons are recently being developed to solve issues of worker injuries and prevent musculoskeletal disorders [1,2]. In particular, there has been a lot of development of spinal exoskeletons to help alleviate the problems of low-back pain, and shoulder exoskeletons to assist users in overhead working scenarios [3,4,5]. These exoskeletons exist predominantly in two categories: passive and active. Active exoskeletons can add energy to the human motion using various types of actuators, ranging from hydraulic, electric, pneumatic or a combination of these [6,7,8]. However, active exoskeletons usually require a substantial energy storage that increases the weight of the system, and have a limited amount of operational time. The additional weight of the exoskeleton increases the inertia of the system which is difficult to compensate [9]. On the other hand, there are passive exoskeleton systems that only use passive viscoelastic elements such as springs and dampers to provide assistance to the user. Even though these devices do not add energy to the human, they have been proven to effectively reduce muscular activity and fatigue, which can alleviate injuries or lower the risk of musculoskeletal disorders [10,11,12,13]. Nevertheless, they usually suffer from a limited versatility as they can restrict some of the user movement for tasks that do not require support, such as walking, for spinal exoskeletons [14] and arm motion for shoulder exoskeletons.

One way of solving this issue is by using passive elements to provide the supporting torque and small actuators to engage or disengage this support making these devices quasi-passive. Actuators used for such purposes are called Clutched Elastic Actuators (CEA) [15]. By means of having the possibility to engage or disengage the passive support of the exoskeleton, unobtrusive movement of the user can be achieved for tasks that do not require assistance. For all other tasks, the support is engaged. One of the problems with such devices is the need for a controller that must determine when to turn the support on and off.

Work regarding the control of devices implementing CEAs is sparse as it is usually limited to the low-level control of forces and stiffness [15,16,17]. Most papers regarding control of exoskeletons, deal with active devices [18,19,20]. Some studies were dealing specifically with controllers for active spinal exoskeletons, where various techniques of lifting were being classified and based on this, appropriate support was given to the user [19,21]. These studies focused on the detection and classification of the upward lifting motion and neglected the initial part, when the body bends down towards the load. This is appropriate only if the exoskeleton can actively generate mechanical power. For a quasi-passive exoskeleton this is not a feasible approach, since the energy to assist the upward motion needs to be stored in the viscoelastic elements already when performing the downward motion. This is a more difficult task as some movements start similarly and only become different in later stages (e.g., sitting down on a chair vs. bending down to lift an object from the ground). Arguably this would imply that the initial part of the motion should be considered the same for both tasks (e.g., bending down is common to both sitting down and lifting an object). This would, however, require a more complex acquisition of the ground truth information, such as video analysis of the performed movements. In the scope of this work, we used clean and elementary ground truth information about the performed movements in combination with a probabilistic classification algorithm, to develop a controller that is able to handle the similarities between the movements and provide a control output for the quasi-passive spinal exoskeleton.

A variety of classification techniques exist that were used for human movement classification from body mounted sensors. These range from simple algorithms like threshold-based classification, k-nearest neighbors, decision trees, to probabilistic approaches such as Gaussian mixture models, hidden Markov models, or support vector machines, fuzzy logic and artificial neural networks [22]. Regarding exoskeleton control, Gaussian mixture models (GMM) have already been implemented for EMG and EEG signal classification for the purpose of controlling an exoskeleton [23,24,25]. GMM are also an effective tool for probabilistic classification of user movements [26,27]. The main advantage of GMM is that they provide a probability estimate for classification rather than assigning new input data to one specific class. As such, the classification of the model is not discrete, which allows us to use this information to condition the transitions of a state machine in order to modulate the control output for the quasi-passive spinal exoskeleton. In this paper, we present an exploitation of the GMM probabilistic classification expanded with a state machine to control the quasi-passive exoskeleton in such a way that it supports the user when needed and is unobtrusive when the support is not necessary.

## 2. Materials and Methods

### 2.1. Experimental Setup

**Figure 1:**
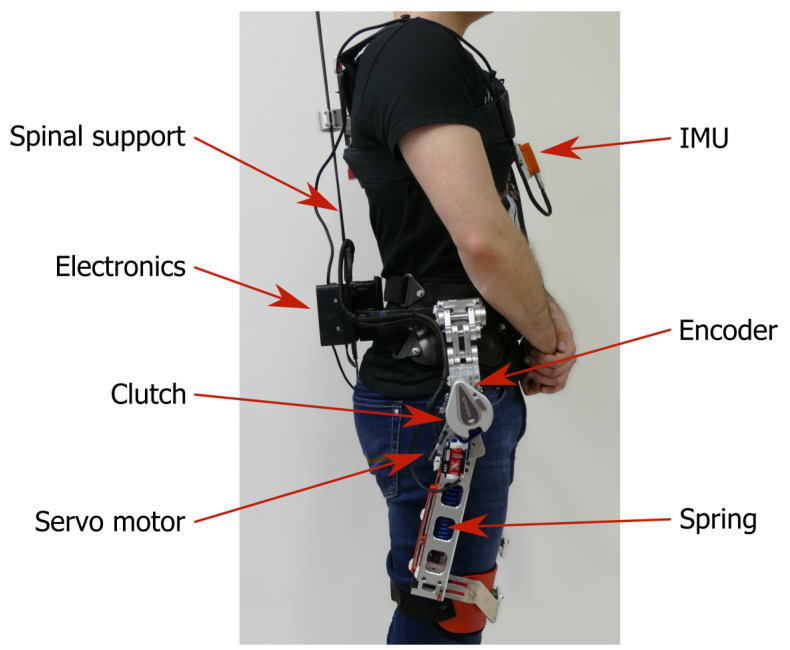
Lateral view of a subject wearing the exoskeleton. Red arrows indicate the locations of key components of the device.

We used a quasi-passive spinal exoskeleton [28] (Figure 1) that can provide support to the subject by unloading the spine when performing lifting movements. It is comprised of two main components: the upper spinal module and the bottom spring mechanism. The upper part is a spinal elastic module with carbon fibers beams that transfers the extension torque generated by the passive spring mechanism at the hip to the trunk of the subject. Additionally, it provides passive support when the subject is performing a trunk flexion. In the bottom part of the exoskeleton there is a spring mechanism that is compressed during hip flexion of the subject which generates an extension torque at the hip. The mechanical design is presented in greater detail in the work of Näf et al. [28]. The spring mechanism can be engaged or disengaged via a clutch located at both hip joints. If the clutch is disengaged, the springs do not provide any support as the mechanism freely rotates around the hip joint. This means that the exoskeleton does not hinder the subject when performing movements like walking or sitting down. It is important to note that the mechanical design of the clutch enables a transition from engaged to disengaged or vice versa only when the forward rotation around the hip joint of the exoskeleton is lower than 25° The rotation around the hip joint of the exoskeleton is directly correlated with the subject’s hip angle.

To automatically engage or disengage the clutch, one small servo motor (KST X15-908, KST Digital Technology Limited, Meihzou, Guangdong, China) is used on each side of the exoskeleton. Each servo motor is attached to the lever arm of the clutch and can engage or disengage the clutch in 0.05 s. For a regular motion of the user, the expected rotational velocity of hip flexion is around 100${}^{\circ }$/s. Based on the servo actuation timing, the subject can move for 5${}^{\circ }$ during the activation of the clutch. This limits the actual engagement window of the clutch down to 20${}^{\circ }$ of hip flexion.

The exoskeleton is equipped with sensors to monitor the configuration of the exoskeleton and therefore the pose of the subject wearing it. It has built-in rotational encoders in both hip joints to monitor the hip flexion or extension (AS5048A, ams AG, Premstaetten, Austria). In addition to the encoders, an IMU (Xsens, Enschede, the Netherlands) is attached on the chest straps of the exoskeleton. The IMU provides information of the orientation of the subject’s trunk in space. For the experiment performed in this study, we recorded the orientation of the subject’s trunk in the sagittal plane.

The exoskeleton includes on-board electronics to read sensor data and send commands to the servo motors. The main computing module consists of a Raspberry Pi Model 3B+ (Raspberry Pi Foundation, Cambridge, UK). It allows the programming of a controller in MATLAB Simulink (Mathworks, Natick, MA, USA) which deploys the compiled code on the target hardware. The power supply for the raspberry Pi is an off-the-shelf product PiJuice HAT (Pi Supply, East Sussex, UK). It provides uninterrupted power supply for the Raspberry Pi from a 4.2 V, 1820 mAh battery. The servo motors are powered from a separate 7.4 V, 1000 mAh Li-Poly battery.

### 2.2. Experimental Protocol

Seven healthy subjects participated in the study (age: 27.14±2.12 years, height: 172.00±9.27 cm, weight: 69.86±12.69 kg). The experiment was conducted at Jožef Stefan Institute in accordance with the principles stated in the Declaration of Helsinki and approved by the Slovenian National Medical Ethics Committee (No. 339/2017/7).

**Figure 2:**
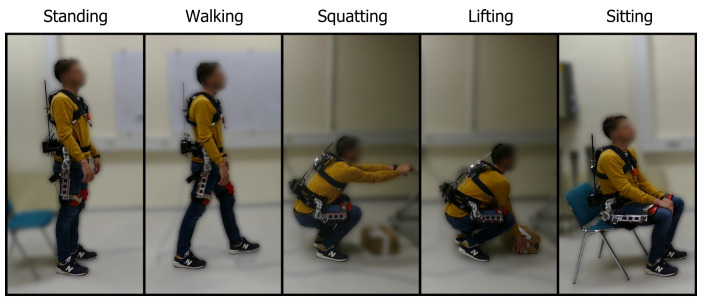
Example of performed tasks during the experiment.

Subjects performed a sequence of tasks while wearing the exoskeleton, representative of a typical working environment, shown in Figure 2. The task sequence was predetermined and consisted of random-number repetitions of the following movements: walking, sitting, squatting and lifting a 3 kg box from the floor. In between every movement, the subjects came back to a normal upright stance and awaited instructions on the next movement. The sequence of tasks was unknown to the subjects and the instructions were given out verbally, only after every completed movement. Each subject performed in total 20 walks, 20 squats, 20 box lifts and 20 chair sits. The subjects were not instructed to perform any specific lifting technique such as stoop or squat lifting. They were free to use their preferred lifting technique as well as perform all movements at their preferred speed. Data from the sensors was recorded using the Raspberry Pi at a frequency of 100 Hz. For reference, the average duration of the performed movements was 1.38 s ± 0.73 s, 4.64 s ± 0.86 s, 2.67 s ± 0.47 s, 2.98 s ± 0.39 s and 5.61 s ± 1.57 s for the tasks of standing, walking, squatting, lifting and sitting, which corresponds to an average of 138, 464, 267, 298 and 561 data samples. The total length of the experiment was on average 460.5 ± 52.3 s. Prior to the start of each recording session, we recorded the orientation of the IMU for 1 s. The mean value of this recording was used as a reference for normal upright standing (0${}^{\circ }$ of trunk inclination). During the experiment, the exoskeleton clutch was disengaged at all times.

To have a ground truth label to train and validate our classification method, the recorded dataset was segmented and hand-labeled. The set of possible labels was: 1—standing, 2—walking, 3—squatting, 4—lifting, 5—sitting. Labeling was based on the set of instructions given to the subjects during the experiment. For example, the whole motion of performing the instruction “Sit”, “Stand up”, was labeled as sitting. The start and end of the task were determined by the standing phase.

### 2.3. Classification Algorithm with GMM

The foundation of our control algorithm is based on GMM to discriminate between the various tasks performed by the subject. This is done in two steps: fitting and predicting.

First, data belonging to each task *j* was modelled using a superposition of K Gaussian densities (Gaussian mixtures) by

(1) $$p_{j}\left(x\right)=\sum_{k=1}^{K}\tau_{k}\phi_{k}\left(x|\mu_{k},\Sigma_{k}\right),$$

where x is a 6×1 vector of input data (right encoder angle, left encoder angle, IMU orientation in the sagittal plane, right encoder angular velocity, left encoder angular velocity, rate of change of IMU orientation in the sagittal plane). Every *k*-th Gaussian density denoted as $\phi_{k}\left(x|\mu_{k},\Sigma_{k}\right)$ is a component of the mixture and has its own mean $\mu_{k}$ and covariance $\Sigma_{k}$. $\tau_{k}$ are the mixing coefficients of the components. Both $p_{j}\left(x\right)$ and the individual Gaussian components are normalized such that:

(2) $$\int_{-\infty }^{\infty }p_{j}\left(x\right)=1$$

and

(3) $$\sum_{k=1}^{K}\tau_{k}=1.$$

**Figure 3:**
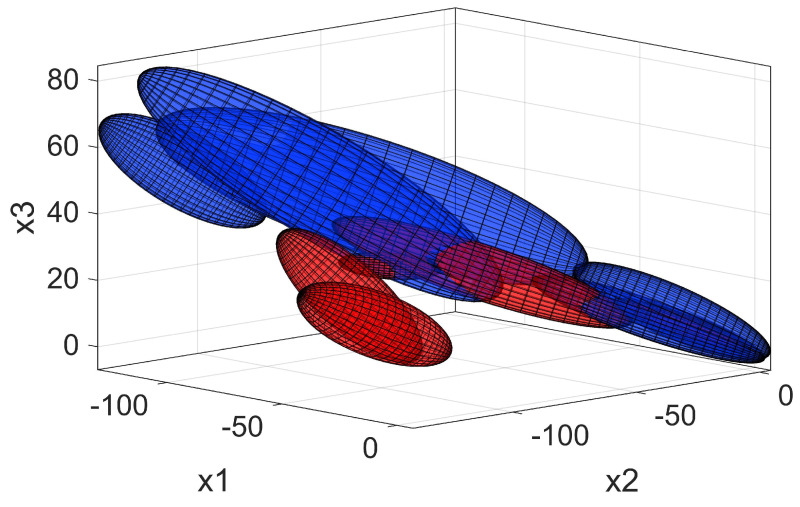
GMM representation in a reduced feature space for the lifting (blue) and sitting task (red); $K_{lift}=5$, $K_{sit}=5$. The axis $x_{1}$, $x_{2}$ and $x_{3}$ represent right hip angle, left hip angle and trunk inclination, respectively. For reference, all movements start in the bottom right corner, continue towards the left side of the graph and come back to the starting position.

To fit the Gaussian mixtures, we used the Expectation Maximization algorithm for fitting a predefined number of mixtures to the selected data. The number of mixtures *K* used for fitting was set to 1 for the standing task. For the tasks of walking, squatting, lifting and sitting, the value *K* was determined in an iterative process [29], as follows. For each model with *K* Gaussians used, the Bayesian information criterion (BIC) was calculated and compared to the value of the previous model (K−1). If the new value of BIC was lower, the new model was kept, and the iteration continued until the value did not lower anymore or the set maximum value (K=5) was reached. In Figure 3 we present an example of the trained GMM in a reduced feature space for the lifting and sitting task.

To use the Gaussian mixture models for classification of new input data we calculate the posterior probability of the new input data x belonging to each task *j*:

(4) $$p_{j}\left(x\right)=\sum_{k=1}^{K}\tau_{k}\left(\frac{e^{-\frac{1}{2}{\left(x-\mu_{k}\right)}^{T}\Sigma_{k}^{-1}\left(x-\mu_{k}\right)}}{\sqrt{{\left(2\pi \right)}^{D}\left|\Sigma_{k}\right|}}\right),$$

where *D* is the dimensionality of the model (6 in our case).

We can join these values in a vector p(x) that contains the posterior probabilities for each task. At this point it is possible to compare all the probability values with each other and select the task with the highest probability. To do this, we define a function imax(p) that selects the index *j* in the vector p that has the maximum probability,

(5) $$imax\left(p\right)=\left\{j\;|\;p_{j}=max\left(p\right)\right\},$$

which gives us the prediction to which state the new input data x belongs to. This approach already provides valid and useful information about any new input data into the model. However, this can be problematic when GMM of different tasks overlap. Such an overlap can be observed in the bottom right corner of Figure 3. This is an outcome of the protocol used for acquiring the ground truth that was used for training the model. In this case, the prediction using imax(p) is not stable as it can change from one task to another for every new data sample acquired. To overcome this limitation and preserve the clean acquisition of ground truth, we developed a state machine controller that is presented in SubSection 2.4.

### 2.4. Integration of GMM with a State Machine

**Figure 4:**
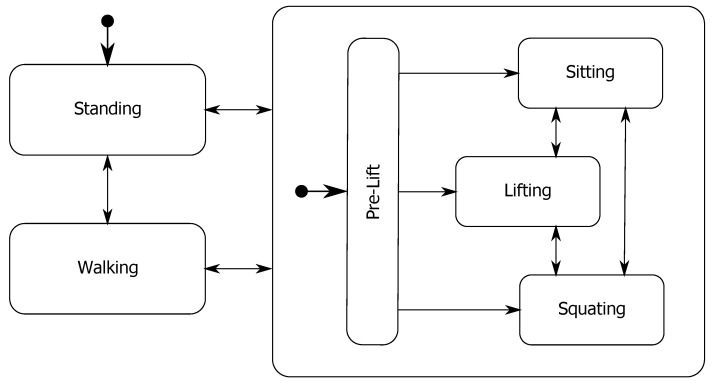
Structure of the state machine controller with all possible transitions between states. The states of pre-lift, squatting, lifting and sitting are grouped inside a superstate which allows transitions back to the walking or standing state. Arrows with circles denote initial transitions.

To complement the GMM probability outputs we developed a state machine controller which is schematically presented in Figure 4. The states of the controller are the five tasks performed during the experiment (standing, walking, squatting, lifting and sitting) along with a *pre-lift* state. We added a *pre-lift* state, because of the similarity of the models in the early stages of performing the tasks of squatting, lifting and sitting. By using this state, we conditioned the output of the state machine to classify a *pre-lift* until the probability of squatting, lifting or sitting reaches a high enough value. This limits the amount of transitions between states in the initial part of the downward movement when the subject starts bending down.

The transitions between the states are conditioned by probabilities of p(x), normalized based on the sum of probabilities of all tasks (J=5) for the current input x,

(6) $$p^{n}\left(x\right)=\frac{p(x)}{\sum_{j=1}^{J}p_{j}\left(x\right)}.$$

With this normalization, we simplify the comparison of probabilities for the current input x. However, the normalized probabilities, do not include information of the actual value of the posterior probability of x belonging to a task *j*. This is important, as it defines whether the current input data is similar to the data used for training the model or not. To account for this, we compare the current probability value of each state $p_{j}\left(x\right)$ with the maximum probability of the model for that state:

(7) $$m_{j}\left(x\right)=\frac{p_{j}\left(x\right)}{max(p_{j}\left(x\right))}.$$

If the current probabilities for all states are lower than 0.005 of their respective maximum, the transitions between the states in the state machine are disabled. The initial state of the state machine is denoted by the circle and arrow leading to the state standing. The transition to standing occurs, when the sensors are calibrated in a normal standing pose of the user. After this, the state machine controller can switch between all the different states based on the following transition rules. The transition rule from standing or walking to the *pre-lift* state is defined as:

(8) $$p_{3}^{n}\left(x\right)+p_{4}^{n}\left(x\right)+p_{5}^{n}\left(x\right)>h_{1}.$$

All other transitions in the state machine from a current state *i* to the next state i+1 are defined as

(9) $$p_{i+1}^{n}\left(x\right)>h_{1},$$

where $h_{1}$ is a fixed threshold value set to 0.8. Additionally, we add a hold condition that holds the current state of the state machine when the subject is in the middle of a sit, squat or lift. The rule for the hold condition is $abs\left(v_{T}\right)<v_{1}$ AND ($p_{3}^{n}\left(x\right)>h_{2}$ OR $p_{4}^{n}\left(x\right)>h_{2}$ OR $p_{5}^{n}\left(x\right)>h_{2}$), where $h_{2}$ and $v_{1}$ are fixed threshold values of 0.5 and 5${}^{\circ }/s$ respectively and $v_{T}$ denotes the rate of change of orientation of the subject’s trunk. The hold condition ends when the probability of standing or walking satisfies Equation (9).

### 2.5. Performance Evaluation of the Controller

We evaluated the performance of our controller for task recognition and for the activation of support provided to the user. The methodology used to evaluate task recognition is presented in Section 2.5.1. Activation of support provided to the user was evaluated based on the control signals generated for clutch actuation which is presented in Section 2.5.2.

#### 2.5.1. Evaluation of Task Recognition

To evaluate the performance of task recognition, we performed a leave-one-out cross-validation procedure on the dataset. Data of all except one subject was used to train the GMM and the remaining subject’s data was used as the test dataset. This was repeated for each subject, using every subject once as the testing dataset. To emphasize the importance of the state machine we calculated the performance metrics for the case of using only GMM and the combination of GMM with the state machine (GMM+S). First we looked at the overall accuracy, sensitivity and specificity of classification for GMM+S considering all input data samples collected during the experiment. We defined these classification metrics as:

(10) $$Accuracy=\frac{TP+TN}{TP+TN+FP+FN}\;,$$

(11) $$Sensitivity\left(k\right)=\frac{TP_{k}}{TP_{k}+FN_{k}}\;,$$

(12) $$Specificity\left(k\right)=\frac{TN_{k}}{TN_{k}+FP_{k}}\;,$$

where TP, TN, FP, FN denote the number of true positives, true negatives, false positives and false negatives. For the calculation of specificity for the 5 states, the TN and FP values were calculated for a one vs. all comparison.

More important in our case, are classification results for binary conditions of support ON and support OFF. In this case, we define a true positive result (TP) as a classification of support ON for lifting or squatting movements. A true negative result (TN) is considered a classification of support OFF for standing, walking and sitting movements. The definitions of the performance metrics of accuracy, sensitivity and specificity remain the same as defined in Equations (10)–(12). Since the calculation of these metrics across all input data is too general, we additionally calculated their values over the course of the performed movements. To do this, we normalized each individual repetition of a task over time. We then calculated the performance metrics for each 5% of the normalized movement trajectories for each subject. The final results are presented with the mean and standard deviation of the performance metrics across all the test subjects.

#### 2.5.2. Evaluation of Support Activation

To evaluate the efficacy of our controller for support activation we performed a simulation of the actuation of the exoskeleton’s clutch. As stated in Section 2.1, the clutch of the exoskeleton needs some time to actuate. We have taken into account this actuation time, by reducing the angle at which successful engaging of the clutch is deemed possible. For an average motion of the user, this angle corresponds to 20${}^{\circ }$. The exoskeleton should support the lifting and squatting movements, therefore the clutch needs to successfully engage at the beginning of these movements. In contrast to this, the clutch should be disengaged for the standing, walking and sitting tasks to allow free movement to the user. The results of this evaluation are presented based on the binary state of the exoskeleton’s clutch. A true positive result (TP) is considered a successfully engaged clutch for lifting and squatting movements. A true negative result (TN) is considered a disengaged clutch for standing, walking and sitting movements.

## 3. Results

### 3.1. Task Recognition

Using GMM+S, the overall recognition accuracy of all 5 tasks (standing, walking, squatting, lifting, sitting) for all input data samples was 81.26±2.89% (mean ± s.d.). The average sensitivities across 7 subjects for standing, walking, squatting, lifting and sitting were 92.82±5.38%, 91.09±3.15%, 65.42±24.34%, 89.29±6.60%, 67.54±3.02% respectively. Whereas the specificities were: 78.83±3.78%, 77.70±2.99%, 84.82±4.78%, 88.39±3.12%, 76.67±3.69%.

**Figure 5:**
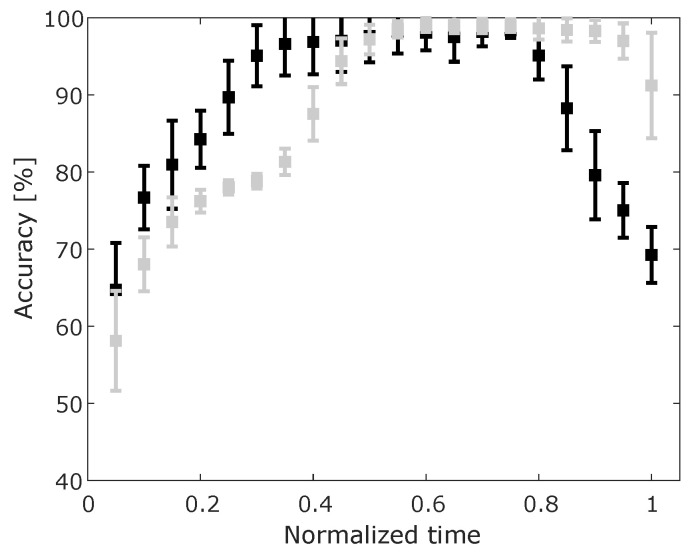
Mean and standard deviation of accuracy over the course of the performed movement for ON/OFF classification. Using only the maximum GMM probability (black) and using GMM+S (grey).

However, in our case, it is more important to look at results of support ON vs support OFF classification. For this binary case of support ON/OFF, the overall classification accuracy for all input data samples was 88.57±0.94% and the sensitivity and specificity were 95.72±2.62% and 86.23±1.57% respectively. The results of the performance metrics of accuracy over normalized trajectories are presented in Figure 5. We can observe that there is a very high accuracy for both cases (GMM and GMM+S) in the middle of the movement (at 0.5 of the normalized time). The GMM only (black error bars) shows a higher accuracy of recognition at an earlier stage of the movement (accuracy > 90% at normalized time 0.3).

**Figure 6:**
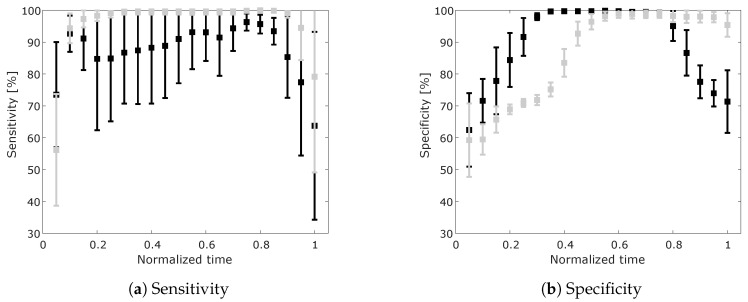
Mean and standard deviation of sensitivity (**a**) and specificity (**b**) over the course of the performed movement for ON/OFF classification. Using only the maximum GMM probability (black) and using GMM+S (grey).

Sensitivity and specificity for ON/OFF classification are presented in Figure 6a,b. Here we can observe that using GMM only we obtain higher specificity of classification in the first half of the performed movements. This indicates a higher success rate of classifying support OFF when no support is required. However, the sensitivity of classification using GMM only is lower, with a higher standard deviation, compared to using the combinations of GMM with the state machine (GMM+S). This means that using the approach of GMM+S, we can reduce the number of misclassified movements when the support of the exoskeleton is required.

### 3.2. Support Activation

**Figure 7:**
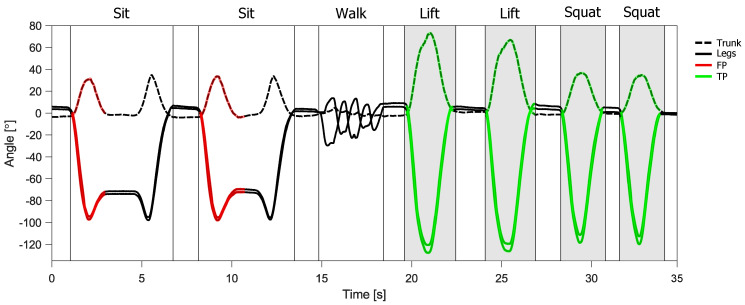
Example of control output for the support of the quasi-passive spinal exoskeleton. Shaded grey areas indicate when the support needs to be active. Highlighted trajectories represent the real-time output of the controller for support ON state. True positive activation of the support is denoted in green, False positive activation is denoted in red. Graph labels for the sections of standing are omitted for clarity.

An example of the control outputs of the controller is presented in Figure 7. Lift and squat movements, when the support of the exoskeleton is required, are highlighted with shaded grey areas. The rest of the data represents sitting, standing and walking. In this example, all the movements requiring support were correctly classified and the support was provided successfully, which is denoted with a green highlight. Both sitting tasks are initially misclassified and are therefore highlighted in red. The results of support activation for all movements and for all subjects are presented in the form of a confusion matrix in Table 1. Accuracy of support activation was 86.72±0.86% (mean ± s.d.). The sensitivity and specificity of support activation were 97.46±2.09% and 83.15±0.85% respectively. The specificity is reduced, due to the many sitting tasks being supported in the early part of the movement as can be seen in the example presented in Figure 7, highlighted in red. However, it is important to note that 95.05±4.95% of sitting tasks were correctly classified in the middle of the movement.

## 4. Discussion

We described our approach of using probability outputs of a GMM in combination with a state machine to generate control signals in real time for a quasi-passive spinal exoskeleton device. The inputs to the controller were limited only to the sensors embedded in the device. With this approach we achieved a very high accuracy of task recognition in the middle of the movement, when the support to the user is most critical for lifting movements. These results indicate that our approach would be suitable also for active exoskeletons, where this method could be used to select different types of support profiles for various lifting techniques. Additionally, based on the type of lifting technique and an a priori knowledge of joint loading for each movement, the exoskeleton could prevent human joint overloading, while still enabling a seamless execution of the task [30].

Using the state machine, we complemented the versatility of GMM by controlling transitions between states during downward motion. With this, we achieved very good results with a sensitivity of support activation of 97.46±2.09%. The specificity of support activation was only 83.15±0.85%, which is mainly due to the many sitting tasks being initially misclassified. Due to the similarity between lifting and sitting movements at the beginning of the motion (Figure 3), it is difficult to disengage the support for sitting without compromising support for lifting. In this case, a more feasible solution would be the use of a different clutch mechanism that can be disengaged under load. Nevertheless, classification errors are inevitable and can greatly compromise the comfort or safety of the wearable device. Striving for reducing these errors is important, but equally so is the implementation of additional safety measures that prevent injuries or discomfort even in the unlikely event of misclassified user movements or intentions.

The protocol used for determining the ground truth required to learn the GMM, could be easily integrated in the wearable device itself, requiring user input for movement type along with the start and end of movement. In our approach, the absolute values of GMM probability were used only to limit transitions in case of uncertainty (low $m_{j}\left(x\right)$ values). However, this information could also be used to detect movements new to the model or give feedback to the user, prompting an update of the learned model. Another possible extension could be to look at the repetitions of the same movement to influence future predictions.

Even though much of the recent technological advances in spinal exoskeleton control are focused on active devices [31], we believe that the emerging passive and quasi-passive versions are equally important. The many benefits of passive devices are often limited by their poor versatility or the discomfort imparted to the users for performing some tasks [32]. Therefore, the development of hybrid style devices along with adequate control is very important to advance the field of spinal wearable devices. Despite some limitations of this study, we believe our approach proved to be a promising tool for control of quasi-passive spinal exoskeletons and thus provides a meaningful contribution to the state of the art.

## Figures and Tables

**Table 1:** Confusion matrix (mean ± s.d.) for support activation of exoskeleton.

		Required Condition	
		Support ON	Support OFF
Output	Support ON	97.46±2.09	16.85±0.85
	Support OFF	2.54±2.09	83.15±0.85
