# Erratum: Jamšek et al. Gaussian Mixture Models for Control of Quasi-Passive Spinal Exoskeletons. *Sensors* 2020, *20*, 2705

**DOI:** 10.3390/s21217359

**Published:** 2021-11-05

**Authors:** Marko Jamšek, Tadej Petrič, Jan Babič

**Affiliations:** 1 Laboratory for Neuromechanics and Biorobotics, Department of Automation, Biocybernetics and Robotics, Jožef Stefan Institute, 1000 Ljubljana, Slovenia; tadej.petric@ijs.si (T.P.); jan.babic@ijs.si (J.B.); 2 Jožef Stefan International Postgraduate School, Jamova cesta 39, 1000 Ljubljana, Slovenia

There was an error in the original article [1]. The variable used for the superscript in the denominator of the equation, denoting the dimensionality of the model, was incorrectly written as K and thus conflicted with the variable denoting the number of Gaussian mixtures. A correction has therefore been made to Section 2.3, Equation (4):

(4) $$p_{j}\left(x\right)=\sum_{k=1}^{K}\tau_{k}\left(\frac{e^{-\frac{1}{2}{\left(x-\mu_{k}\right)}^{T}\Sigma_{k}^{-1}\left(x-\mu_{k}\right)}}{\sqrt{{\left(2\pi \right)}^{D}\left|\Sigma_{k}\right|}}\right),$$

where *D* is the dimensionality of the model (6 in our case).
